# Vaginal microbial shifts are unaffected by oral pre-exposure prophylaxis in South African women

**DOI:** 10.1038/s41598-022-20486-z

**Published:** 2022-09-28

**Authors:** Noluthando Mazibuko-Motau, Parveen Sobia, Jiawu Xu, Joseph Ahmed Elsherbini, James E. San, Lara Lewis, Andile Mtshali, Gugulethu Mzobe, Lungelo Ntuli, Salim S. Abdool Karim, Leila E. Mansoor, Quarraisha Abdool Karim, Douglas S. Kwon, Derseree Archary, Sinaye Ngcapu

**Affiliations:** 1 Centre for the AIDS Programme of Research in South Africa, 2nd Floor, Doris Duke Medical Research Institute, 719 Umbilo Road, Durban, 4041 South Africa; 2 Department of Medical Microbiology, University of KwaZulu-Natal, Durban, South Africa; 3 Ragon Institute of MGH, MIT, and Harvard, Massachusetts General Hospital, Cambridge, MA USA; 4 KwaZulu-Natal Research Innovation and Sequencing Platform, Nelson R Mandela School of Medicine, University of KwaZulu-Natal, Durban, South Africa; 5 Department of Epidemiology, Columbia University, New York City, NY USA; 6 Division of Infectious Diseases, Massachusetts General Hospital, Boston, MA USA; 7 Harvard Medical School, Boston, MA USA

**Keywords:** Microbiome, HIV infections

## Abstract

Vaginal microbiota have been shown to be a modifier of protection offered by topical tenofovir in preventing HIV infection in women, an effect not observed with oral tenofovir-based pre-exposure prophylaxis (PrEP). It remains unclear whether PrEP can influence the vaginal microbiota composition. This study investigated the impact of daily oral tenofovir disoproxil fumarate in combination with emtricitabine for PrEP on the vaginal microbiota in South African women. At baseline, *Lactobacillus iners* or *Gardnerella vaginalis* dominant vaginal communities were observed in the majority of participants. In cross sectional analysis, vaginal microbiota were not affected by the initiation and use of PrEP. Longitudinal analysis revealed that *Lactobacillus crispatus*-dominant “cervicotypes 1 (CT1)” communities had high probability of remaining stable in PrEP group, but had a higher probability of transitioning to *L. iners*-dominant CT2 communities in non-PrEP group. *L. iners*-dominant communities were more likely to transition to communities associated with bacterial vaginosis (BV), irrespective of PrEP or antibiotic use. As expected, BV-linked CTs had a higher probability of transitioning to *L. iners* than *L. crispatus* dominant CTs and this shift was not associated with PrEP use.

## Introduction

Over the past decade, studies have demonstrated the efficacy of prophylactic antiretrovirals (ARVs) for the prevention of HIV, with oral tenofovir-based treatment showing markedly reduced rates of HIV infection among men who have sex with men, transgender women, injection drug users, serodiscordant couples, and heterosexual men and women^1–5^. Pre-exposure prophylaxis^6^ for men using either daily or intermittent dosing strategies has consistently demonstrated protective efficacy ranging from 44 to 96%^1–5,7^. Despite adherence, PrEP for women in Southern Africa has yielded mixed results, underscoring a plausible role for other biological factors that mitigate PrEP efficacy^6,8,9^.

Previous studies have suggested that the vaginal microbiome can significantly modulate the efficacy of topical tenofovir-based PrEP^10,11^, an effect not observed with oral tenofovir-based PrEP^12^. In addition, proteomics analyses of vaginal specimens of participants from the CAPRISA 004 1% tenofovir gel trial showed a significantly reduced risk of HIV acquisition in women with *Lactobacillus* dominant compared to non-*Lactobacillus* dominant microbiota^8,10^. Furthermore, the tenofovir levels were reduced in vaginal specimens from women with a *Gardnerella vaginalis* dominant microbial community, suggesting that the depletion of tenofovir was secondary to drug metabolism by *G. vaginalis*^8,10^. These findings are supported by high tenofovir levels in cervical tissue of women with a Nugent score of ≤ 3 (*Lactobacillus* dominant) and reduced tenofovir levels in women with bacterial vaginosis (BV) (*G. vaginalis* dominant^11^). However, none of these studies have characterized the potential impact of PrEP on the vaginal microbial communities of healthy women in sub-Saharan Africa. To address this gap, we investigated the impact of longitudinal oral tenofovir disoproxil fumarate + emtricitabine (TDF-FTC) PrEP on the vaginal microbiome of healthy South African women.

## Materials and methods

### Study design, participants, and sample collection

This is a retrospective study within the CAPRISA 082^13^ and CAPRISA 084 (https://www.caprisa.org/Pages/CAPRISAStudies) studies. CAPRISA 082 was a prospective observational cohort study of HIV risk factors and prevention choices in young women aged 18–30 years old from an urban and rural population in KwaZulu-Natal, South Africa. CAPRISA 084 was a PrEP demonstration project that assessed the feasibility, acceptability, uptake and patterns of daily oral TDF/FTC PrEP provided as part of sexual reproductive health services to young women and men (aged 18 years and older) at risk of acquiring HIV in eThekwini, Vulindlela and Umlazi in KwaZulu-Natal, South Africa. Women living with HIV and those who had other factors contraindicated for PrEP use were excluded from both studies. Consenting participants in both studies were followed monthly for up to 3 months, then quarterly for approximately 18 months. Some CAPRISA 082 women agreed to take PrEP while others disagreed, and all were reimbursed for attending clinic visits. All CAPRISA 084 participants were provided with PrEP at all visits and were not reimbursed for attending clinic visits. For this study, we only included cervicovaginal swab samples collected at baseline, 3, and 6 months. At each visit, cervicovaginal swabs collected from the posterior fornix and lateral vaginal walls from each participant were tested for vaginal pH using pH indicator strips, and for *Chlamydia trachomatis, Neisseria gonorrhoeae, Mycoplasma genitalium* and *Trichomonas vaginalis* using PCR. Gram staining was performed to confirm a diagnosis of BV based on Nugent score ≥ 7. CAPRISA 082 and 084 participants were treated with regimens recommended in the South African STI treatment guidelines for the following diagnoses: *Chlamydia trachomatis* (azithromycin 1 g oral)*, Neisseria gonorrhoeae* (ceftriaxone 250 mg intramuscular and azithromycin 1 g oral)*, Trichomonas vaginalis* (metronidazole 2 g orally) and candidiasis (clotrimazole 500 mg pessary and clotrimazole 1% cream). Women who had a Nugent score ≥ 4 were treated with metronidazole (2 g oral single dose). At follow-up visits, CAPRISA 082 participants were managed syndromically, however CAPRISA 084 participants were screened and treated for STIs and BV. All participants provided written informed consent. The study was approved by the Biomedical Research Ethics Committee of the University of KwaZulu-Natal (BREC number: BE603/18). All the experimental procedures are in the accordance with the relevant ethical guidance and regulations.

#### Bacterial DNA extraction, 16S rRNA gene sequencing, taxonomic assignment and CTs assignment

Total nucleic acid was extracted using phenol–chloroform from stored vaginal swabs and the V4 region of the 16S rRNA gene was amplified and sequenced using the Illumina MiSeq as previously described^14^. Divisive Amplicon Denoising Algorithm (DADA) 2^15^ was used to filter for quality, trim, and identify inference of amplicon sequence variant (ASV). ASVs were taxonomically classified to genus or higher levels using a Naïve Bayes classification approach^16^ and SILVA ribosomal RNA database^15^. The ASVs for *Lactobacillus, Prevotella, Sneathia* and *Mobiluncus* were further refined to the species level with speciateIT (version 1.0, http://ravel-lab.org/speciateIT). A phyloseq object containing a phylogenetic tree, ASV table, taxonomic table, and sample metadata was created using the phyloseq R package^17^. Based on composition and diversity at species-level where possible and genus-level, bacterial communities clustered into 4 basic groups, which we referred to as “cervicotypes”^14^.

### Statistical analysis

Descriptive statistics for quantitative variables were summarized using medians and interquartile ranges (IQRs) whilst categorical data were summarized with both frequency counts and percentages. Mann–Whitney U test was used to compare continuous variables and Fisher’s Exact test for categorical comparisons. α-diversity including Richness (Chao1), evenness (Simpson’s E) and phylogenetic diversity (Faith’s PD) estimates were calculated using the *R vegan library*. β-diversity between samples was estimated by Bray–Curtis distances. Non-metric Multidimensional scaling (NMDS) was performed to assess differences in taxonomy profiles among samples based on the Bray–Curtis distance of ASV relative abundance using the *metaMDS function* in the *vegan R package*. The transition probabilities are calculated as proportions of the samples (stratified by antibiotic and PrEP use) shifting from one CT to the next. Permutational multivariate analysis of variance (PERMANOVA) was performed with *Adonis* function in the *vegan R package* with a similarity index using 9999 permutations to measure the differences in beta diversity between PrEP and non-PrEP groups. All statistical calculations were carried out using R and SAS version 9.4 (SAS Institute Inc., Cary, NC, USA).

### Data availability

The datasets generated or analysed during the current study are publicly available in the NCBI BioProject respository, (http://www.ncbi.nlm.nih.gov/bioproject/858949) under accession number PRJNA858949.

## Results

### Study participants

A total of 100 women were included in this sub-study, of whom 60 (60%) were from CAPRISA 082 and 40 (40%) from CAPRISA 084. All CAPRISA 084 participants were provided with PrEP. For CAPRISA 082 participants, 24 (40%) were provided with PrEP and the other 36 (60%) were not (Table 1). Most women were below 30 years (median age 24 years (IQR 21.5–28) of age, but compared with non-PrEP group, PrEP group was older [median age 26.5 years (IQR 22.5–34) vs 22 years (20.5–25); *p* = < 0.0001]. Although majority of women reported having a stable partner (84/100, 84%), the proportion of women in the PrEP group was lower than that of non-PrEP group [49/64 (76.6%) vs 35/36 (97.2%); *p* = 0. 0087]. A greater proportion of participants in the PrEP group were married (12/64, 18.8%) compared to those in the non-PrEP group (0/36; *p* = 0.0037). A high number of women in the PrEP group (16/64, 26.2%) reported having a partner who was HIV positive compared to those in the non-PrEP group (0/36; *p* = 0.001). Study site, education level, vaginal sex within 30 days and contraceptive use were similar between PrEP and non-PrEP groups. Depo medroxyprogesterone acetate (49%, 49/100) was the most common contraceptive used in both groups.

**Table 1:** Demographic and behavioural characteristics of study participants at baseline.

Variables		Overall (N = 100)	PrEP group (N = 64)	Non-PrEP group (N = 36)	*p*-value
		n (%)	n (%)	n (%)	
Study	CAPRISA 082	60	24	36	
	CAPRISA 084	40	40	0	
Age (years)	Median (IQR)	24 (21.5–28)	26.5 (22.5–34)	22 (20.5–25)	< 0.0001
	≤ 21	25 (25.0)	10 (15.6)	15 (41.7)	0.0073
	22–25	33 (33.0)	17 (26.6)	16 (44.4)	0.0796
	26–30	22 (22.0)	17 (26.6)	5 (13.9)	0.2086
	31–40	8 (12.5)	8 (12.5)	0 (0.0)	0.0479
	40–68	12 (18.8)	12 (18.8)	0 (0.0)	0.0037
Living site	Urban	36 (36.0)	19 (29.7)	17 (47.2)	0.0878
	Rural	64 (64.0)	45 (70.3)	19 (52.8)	
Education	Less than secondary	30 (30.0)	22 (34.4)	8 (22.2)	0.2581
	Secondary or higher	70 (70.0)	42 (65.6)	28 (77.8)	
	Married	12 (12.0)	12 (18.8)	0 (0.0)	0.0037
Partner status	Stable	84 (84.0)	49 (76.6)	35 (97.2)	0.0087
	Casual	5 (5.0)	4 (6.3)	1 (2.8)	0.6514
	No partner	3 (3.0)	3 (4.7)	0 (0.0)	0.5512
Partner HIV positive	Yes	16 (16.0)	16 (26.2)	0 (0.0)	0.0004
	No	61 (61.0)	34 (55.7)	27 (75.0)	0.0815
	Don’t know	20 (20.0)	11 (18.0)	9 (25.0)	0.4441
Vaginal sex in last 30 days	Yes	80 (80.0)	49 (77.8)	31 (86.1)	0.4282
Contraceptive	None	27 (27)	16 (25.0)	11 (30.6)	0.5954
	Depo-Provera	49 (49.0)	31 (48.4)	18 (50.0)	1
	Oral contraceptive	4 (4.0)	4 (6.3)	0 (0.0)	0.2936
	Nur-isterate	5 (5.0)	4 (6.3)	1 (2.8)	0.6514
	Implant	10 (10)	6 (9.4)	4 (11.1)	0.7438
	IUCD	3 (3.0)	1 (1.6)	2 (5.6)	0.2935
	Other	2 (2.0)	2 (3.1)	0 (0.0)	0.5345

### Prevalence of BV and STIs in PrEP and non-PrEP group

In the 100 participants, three CAPRISA 084 women at baseline, two at 3 months and four at 6 months had missing BV data. For women in the CAPRISA 082 study, BV and STIs data were performed at baseline but not at follow-up visits. At baseline, 31.6% (31/97) of women had intermediate BV (Nugent score 4–6) and 21.4% (21/97) had BV (Nugent score ≥ 7) (Table 2). Compared with PrEP group, there was a trend for non-PrEP group to be more likely to have BV (*p* = 0.0576). The proportion with BV in the PrEP group was 14.8% compared to 36.1% in the non-PrEP group (*p* = 0.0231) At 3 months follow-up, 36.8% (14/38) of CAPRISA 084 women had intermediate BV and two of them 5.3% (2/38) had BV. At 6 months, both intermediate BV rate 22.2% (8/36) and BV rate 16.7% (6/36) were almost the same. For STIs, 12% women (12/100) had *C. trachomatis*, 3.1% (3/98) had *T. vaginalis* and 2% (2/100) had *N. gonorrhoea* at baseline, and no difference was observed between PrEP and non-PrEP group. At the 3-month and 6-month visit, 38 and 37 of those in the PrEP group had STI data, respectively, of which 5.3% (2/38) were infected with *C. trachomatis* at 3 months and remained the same 5.3% (2/38) at 6 months. No women had *T. vaginalis* and *N. gonorrhoea* at 3 months and at follow-up visits (Table 2).

**Table 2:** Prevalence of BV and STIs in PrEP and Non-PrEP groups.

Variables		Overall (N = 100)	PrEP group (N = 64)	Non-PrEP group (N = 36)	*p*-value
		n (%)	n (%)	n (%)	
**Baseline**^**a**^					
Bacterial vaginosis (Nugent score)	No BV (0–3)	45 (46.4)	31 (50.8)	13 (36.1)	0.2063
	Intermediate BV (4–6)	31 (32.0)	21 (34.4)	10 (27.8)	0.6527
	BV (7–10)	21 (21.6)	9 (14.8)	13 (36.1)	0.0231
*Trichomoniasis*	Positive	3 (3.1)	1 (1.6)	2 (5.6)	0.5524
*Gonorrhoea*	Detected	2 (2.0)	2 (3.1)	0 (0.0)	0.5345
*Chlamydia*	Detected	12 (12.0)	6 (9.4)	6 (16.7)	0.3416
**Month 3 follow-up**^**b**^					
Bacterial Vaginosis (Nugent score)	No BV (0–3)	22 (57.9)	22 (57.9)	–	–
	Intermediate BV (4–6)	14 (36.8)	14 (36.8)	–	–
	BV (7–10)	2 (5.3)	2 (5.3)	–	–
*Trichomoniasis*	Positive	0 (0.0)	0 (0.0)	–	–
*Gonorrhoea*	Detected	0 (0.0)	0 (0.0)	–	–
*Chlamydia*	Detected	2 (5.3)	2 (5.3)	–	–
**Month 6 follow-up**^**c**^					
Bacterial Vaginosis (Nugent score)	No BV (0–3)	22 (61.1)	22 (61.1)	–	–
	Intermediate BV (4–6)	8 (22.2)	8 (22.2)	–	–
	BV (7–10)	6 (16.7)	6 (16.7)	–	–
*Trichomoniasis*	Positive	0 (0.0)	0 (0.0)	–	–
*Gonorrhoea*	Detected	0 (0.0)	0 (0.0)	–	–
*Chlamydia*	Detected	2 (5.3)	2 (5.3)	–	–

^a^Three participants missing BV data and two missing *Trichomoniasis* data at baseline, ^b^Two missing BV, *Trichomoniasis, Gonorrhoea* and *Chlamydia* results at 3 months, ^c^Four missing BV and *Trichomoniasis* and three missing *Gonorrhoea* and *Chlamydia* results at 6 months. ^b & c^ STI follow-up data not collected in CAP082.

### Composition of the vaginal microbiota at baseline

**Figure 1:**
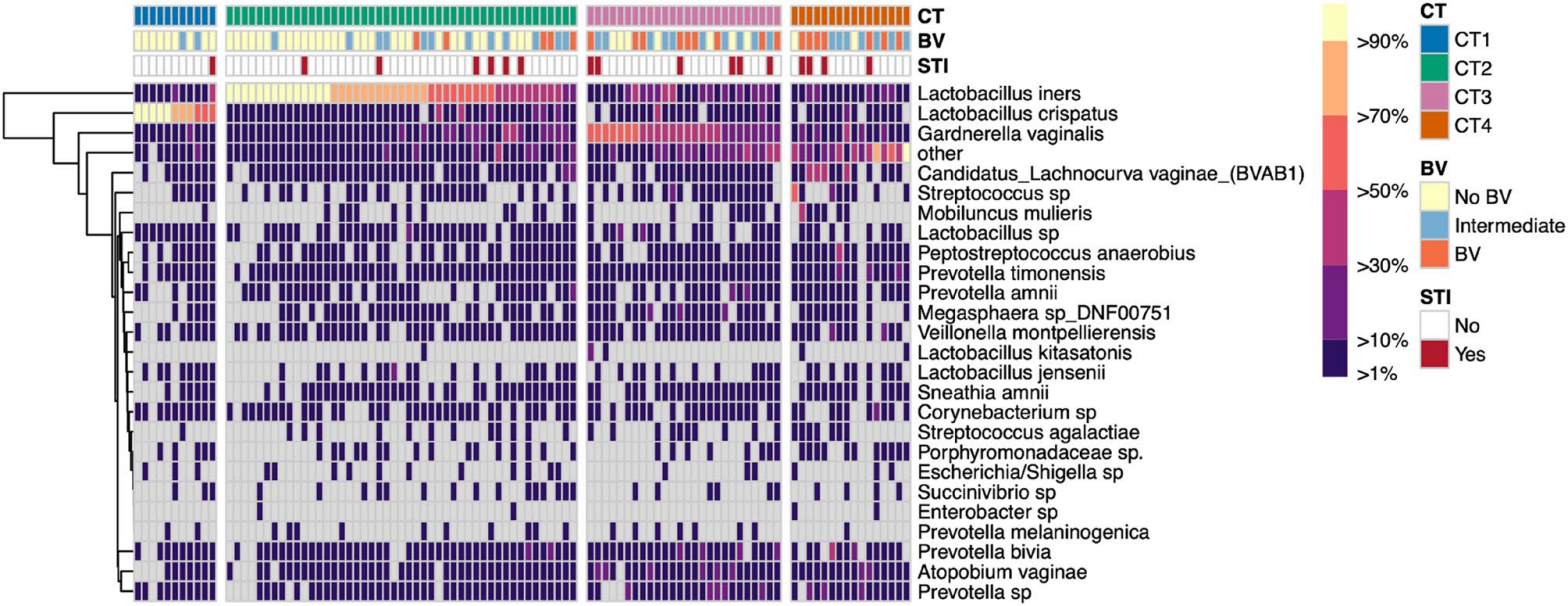
Heat map of the most abundant vaginal bacteria identified by 16S rRNA sequencing in 100 women at baseline. Relative abundance of key taxa across all of the samples was used to determine each of the four CTs: *L. crispatus* (CT1), *L. iners* (CT2), *G. vaginalis* (CT3), and mixed communities not dominated by other taxa (CT4). BV and STI statuses were also depicted.

Based on the composition and relative abundance of bacterial species, we assigned bacterial communities into four CTs (CT1-4, Fig. 1)^14,18^. Samples with the relative majority of sequences (≥ 50%) assigned to non-*iners Lactobacillus* species were defined as CT1 and more than > 97% were *Lactobacillus crispatus*. The communities that had the highest relative abundance of *Lactobacillus iners* and *Gardnerella vaginalis* were classified as CT2, and CT3, respectively. CT4 had a mixed dominant bacterial taxon. Both PrEP and non-PrEP groups had similar vaginal microbial composition at all timepoints (Supplementary Fig. 1). At baseline, 11% (11/100) of women were assigned to CT1, 47% (47/100) to CT2, 26% (26/100) to CT3, and 16% (16/100) to CT4. Of women with intermediate BV (Nugent Score 4–6) and BV (Nugent Score > 7), 18% (2/11) were found in CT1, 36% (17/47) in CT2, 73% (19/26) in CT3 and 88% (14/16) in CT4 (Fig. 1); X^2^ (3, N = 100) = 21.395, *p* = 0.871. Of women with STI infection, 10% (1/11) were found in CT1, 13% (6/47) in CT2, 23% (6/26) in CT3 and 25% (4/16) in CT4; Fisher’s Exact test *p* = 0.568.

### Similar cervicotype composition and transition patterns among groups

**Figure 2:**
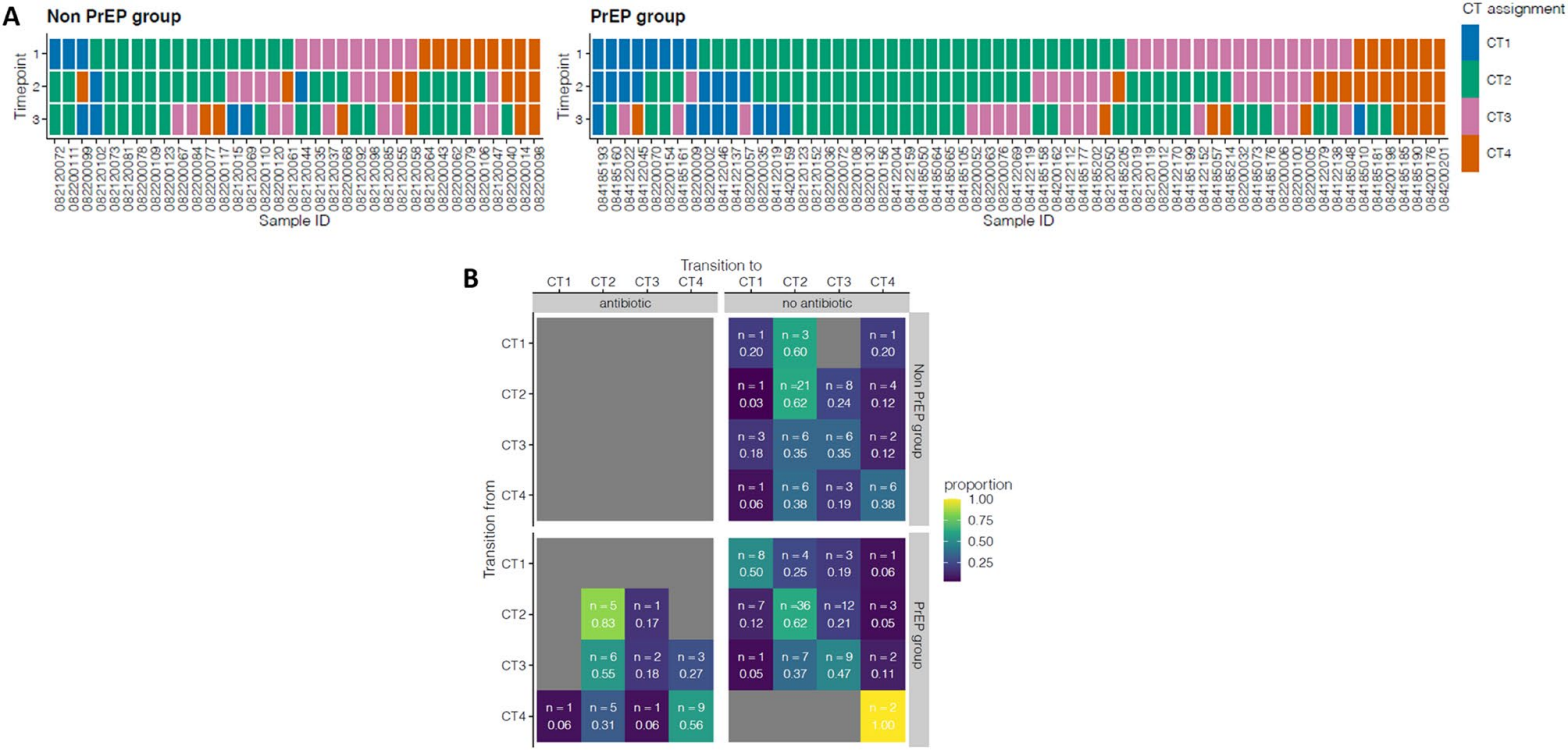
Composition and transitions between cervicotype in PrEP (n = 64) and non-PrEP groups (n = 36). (**A**) Box plots showing CTs assigned to individual participants over 3 visits at baseline, 3 months and 6 months. Blue colour depicts CT1, green CT2, pink CT3, and orange CT4. PID number that starts with 082 refers to women enrolled in CAPRISA 082 and 084 refers to CAPRISA 084 cohort. (**B**) Transition probabilities between CT’s from one visit to another in PrEP and non-PrEP groups who did not use or used antibiotics during the study.

We stratified the composition and structure of vaginal microbiota by PrEP use overtime (Fig. 2A). At baseline, among *L. crispatus* dominated CT1, 8% (3/36) and 13% (8/64) were non-PrEP and PrEP groups, respectively. Of women who were CT1 at baseline, 38% (3/8) transitioned to CT2 and 13% (1/8) to CT3 in the PrEP group while 67% (2/3) non-PrEP group transitioned to CT2 and 33% (1/3) to CT4 at the next time point. In addition, of the four women in the PrEP group who remained stable at 3 months, three women (38%, 3/8) transitioned to new CTs at 6 months. The proportions of *L. iners* dominated CT2 were different between PrEP (n = 32) and non-PrEP (n = 15) groups at baseline; X^2^ (1, N = 45) = 0.188, *p* = 0.665. At 3 months, 34% (11/32) of women with *L. iners* dominated CT2 in the PrEP group transitioned between CTs, with 13% (4/32) to CT1, 19% (6/32) to CT3 and 3% (1/32) to CT4] and 6 non-PrEP group transitioned to new CTs [7% (1/15) CT1, 27% (4/15) to CT3 and 7% (1/15) to CT4]. Furthermore, women with stable CT2 over two visits transitioned to new CTs at 6 months; PrEP group [14% (3/21) transitioned to CT1 and 24% (5/21) to CT3] and non-PrEP group [22% (2/9) to CT3 and 22% (2/9) to CT4]. More than a third of PrEP (33%, 8/24) and non-PrEP (50%, 9/18) groups with BV-associated at baseline transitioned to *Lactobacillu*s dominated CTs at 3 months. PrEP (46%, 6/13) and non-PrEP (50%, 3/6) groups with stable BV-associated CTs over two visits transitioned to *Lactobacillu*s dominated CTs at 6 months. No major shifts in microbial communities were detected in PrEP compared to non-PrEP group.

We further assessed the transition probabilities between CTs in PrEP and non-PrEP groups, respectively (Fig. 2B). CT1 had an increased probability of remaining stable (0.5) in PrEP groups while were more likely to transition to CT2 (0.6) in non-PrEP group. In both groups, those with CT2 had a higher probability of remaining (0.62, respectively) compared to those with other CTs. CT3 in PrEP group without using antibiotics were less likely (0.47) to transition while those who used antibiotics were most likely to transition to CT2 (0.55). In PrEP group, CT4 had an increased probability of remaining (0.56 and 1.00 in antibiotic and non-antibiotic users, respectively) rather than transitioning to other states, while favours transitioning to CT2 (0.38) in non-PrEP group. BV-linked CTs had an increased probability of remaining rather than transitioning to *Lactobacillus* dominant CTs and the observed shifts were not associated with PrEP.

### PrEP or antibiotics did not influence spartial segregation

**Figure 3:**
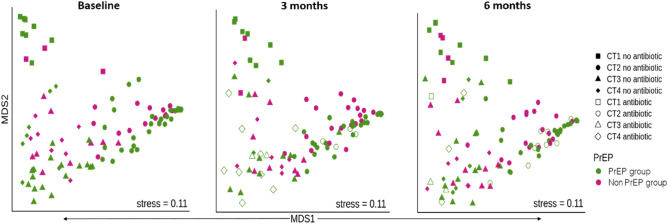
Non-metric Multidimensional scaling (NMDS) of Bray–Curtis showing no clustering dissimilarity of species level relative abundances in PrEP and non-PrEP groups at baseline, 3 months and 6 months. The pink colour represents non-PrEP group and green colour represents PrEP group. Open shape represents participants on antibiotics.

To evaluate the possible impact of PrEP on vaginal microbial communities, we perfomed Non-metric Multidimensional scaling (NMDS) on Bray–Curtis distance at all timepoints. No clustering was detected based on PrEP or antibiotic use (Fig. 3). We further explored the relative contribution of PrEP to the diversity relative to other unknown factors by perfoming a permutational multivariate analysis of variance (PERMANOVA) in adonis. We found no significant differences between the two study groups (PrEP and non-PrEPgroups) at all timepoints (*P* = 0.467, R^2^ = 0.0087, *P* = 0.080, R^2^ = 0.0092 and *P* = 0.344, R^2^ = 0.0108) respectively.

## Discussion

The World Health Organization recommends offering PrEP as an additional prevention choice for persons at increased risk of HIV infection as part of combination HIV prevention approaches^19^. There is a paucity of data on the impact of PrEP on the vaginal microbial communities of healthy women in sub-Saharan Africa. Here, we longitudinally assessed vaginal microbiota in South African women to understand whether PrEP (TDF/FTC) can influence the vaginal microbiome. We found fewer women with a high relative abundance of *L. crispatus* compared to that of *L. iners* and *G. vaginalis* dominant communities. Furthermore, we also observed transitions between CTs in PrEP and non-PrEP groups, with few women with stable *L. crispatus* over time*.*

Studies have revealed that European-American vaginal microbiota have high *Lactobacillus* abundance, while those in women of African descent is more diverse in nature^14,18,20–25^. In addition, Gosmann et al., (2017) demonstrated that 10% of a cohort (N = 236) of South African Black women aged 18–23 years lacked *L. crispatus* dominance in their cervical vaginal microbiome over time^18^. Another study found that only 37% of South African women had *Lactobacillus*-dominant communities^26^. However, these studies solely characterised vaginal microbial communities in women residing in South Africa and no other part of Africa. Therefore, there is a need to understand geographical and lifestyle impact on vaginal microbiome. Consistent with these studies, we demonstrate that only 8%, (3/36, non-PrEP) and 13% (8/64, PrEP group) of women had *L. crispatus-*dominated communities and this number was reduced to one participants (PrEP group) at the 6 month visit. In agreement with previous studies^18,23,26,27^, majority of South African women had vaginal community with high abundance of *L. iners. L. iners*-dominated communities are known to be unstable with high probability of transitioning to a high diversity community state associated with BV. Several factors have been posited to play a dominant role in shaping the vaginal microbiota, including racial lines, vaginal hygiene practices, contraceptive use, sexual behaviour, rectal colonization and host genetics. However, their individual or collective contributions to the shifts in the vaginal microbiome remains unresolved^18,28^.

We explored transition patterns between the observed microbial communities over time. There was no difference in changes in the microbial communities overtime between PrEP and non-PrEP groups. Although *L. iners*-dominant communities were less likely to transition, once they did, they favoured communities associated with BV and increased HIV risk, irrespective of PrEP or antibiotic use. This is consistent with previous studies that showed women with *L. iners*-dominant communities transition more frequently to ecological niche with other bacterial taxa, such as *G. vaginalis, A. vaginae*, and other strict and facultative anaerobes^23,27,29–31^. Furthermore, Munoz et al.^27^ showed that transitions away from *L. crispatus* and towards *L. iners*-dominant communities are less likely to revert to *L. crispatus* communities but most likely to further transition to BV-associated communities. This is particularly concerning and may explain high rates of diverse microbial communities and BV occurrence among women of African descent. These data affirm that there is a microbiome footprint that dictates the colonization of certain bacteria together more strongly, perhaps due to the synergy and mutually beneficial effects for these species to survive together. In addition, viable alternatives such as probiotics that shift transition from *L. iners* to *L. crispatus* leading to curative or preventive vaginal dysbiosis interventions are needed.

We also observed that *G. vaginalis-*dominant communities remained stable over time and more transitions to *L. iners* than *L. crispatus*-dominant communities, irrespective of PrEP or antibiotic use. In this study, some of the PrEP group participants diagnosed with BV were treated with metronidazole at baseline (CAPRISA 082 and CAPRISA 084), 3 months and 6 months (CAPRISA 084 only). Previous studies have demonstrated that BV treatments were associated with temporal changes in the vaginal microbiota^23,32^. Therefore, the observed shifts away from BV-associated communities in this study is more likely attributed to BV treatment rather than PrEP use^23,29,30^*.* This is confirmed by high numbers of observed transitions from BV associated CTs to *L iners* dominated CT in women actively taking antibiotics.

One of the strengths of this study is the longitudinal follow-up and sampling before and after PrEP initiation in at risk South African women. However, this study had limitations which could have influenced the composition of vaginal microbiota. Firstly, PrEP adherence data relied on pill count and self-reporting, and systemic or genital PrEP levels were measured only in a sub-set of women. Secondly, the lack of longitudinal data for STI and BV in the CAPRISA 082 trial that precluded a detailed analyses of the effect of PrEP and BV on the vaginal microbiota. Additionally, we could not measure in detail the impact of BV treatment on vaginal microbiota, instead BV scores (particularly in the CAPRISA 084 cohort- PrEP group) were used as a surrogate of treatment adherence. Furthermore, we did not investigate the impact of other potential co-factors such as STIs, human papillomavirus, herpes simplex virus, hormonal contraceptives, diets, hormonal status, and other vaginal disorders (e.g., aerobic vaginitis), although these may have different biological effects on microbial communities.

In conclusion, our results showed no major differences in vaginal microbial communities between PrEP and non-PrEP groups, suggesting that PrEP initiation does not alter the vaginal microbial communities of HIV-negative sub-Saharan African women. These findings reaffirm that PrEP rollout can be delivered in women with optimal and/or sub-optimal vaginal microbiota. Regular screening for STIs and BV during PrEP care remains an ideal option, particularly in regions with rates of HIV infection.

## Supplementary Information

Supplementary Information.
