# Supplementary material for: Vaginal microbial shifts are unaffected by oral pre-exposure prophylaxis in South African women
Source: Sci Rep. 2022 Sep 28;12:16187. doi: 10.1038/s41598-022-20486-z (PMC9519742; doi:10.1038/s41598-022-20486-z)
Supplement: Supplementary file 1 — Supplementary Information. [file 41598_2022_20486_MOESM1_ESM.docx]

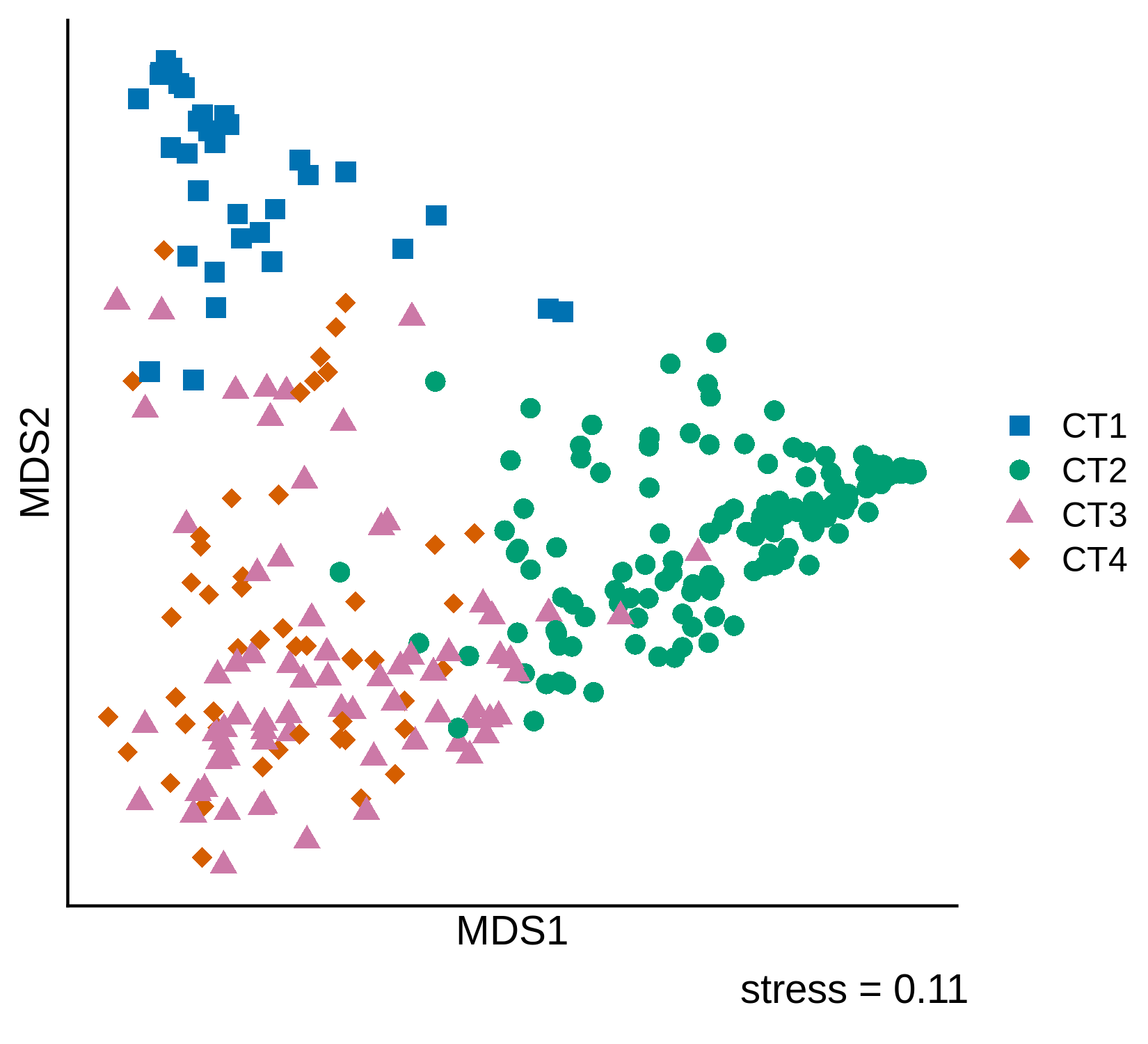


**Figure. S1.** Multidimensional scaling (MDS) of all samples at of all samples at all time points i.e. baseline 3 months and 6 months using Bray Curtis distances of species level relative abundance shows shows clustering of CT1 and CT2 and no distinction between CT3 and CT4. Stress represents the goodness-of-fit statistic that MDS tries to minimize, computed as the square root of the normalized squared discrepancies between interpoint distances in the MDS plot and the smoothed distances predicted from the dissimilarities.
